# Hybrid stage 1 palliation for HLHS: the experience of a tertiary center in a developing country

**DOI:** 10.3389/fcvm.2024.1355989

**Published:** 2024-03-07

**Authors:** Fouad Bitar, Issam M. El-Rassi, Rana Zareef, Yehya Jassar, Jennifer Abboud, Ziad Bulbul, Fadi Bitar, Mariam Arabi

**Affiliations:** ^1^ Department of Pediatrics and Adolescent Medicine, The American University of Beirut Medical Center (AUBMC), Beirut, Lebanon; ^2^ Department of Pediatric Cardiac Surgery, Heart Center of Excellence, Al Jalila Children’s Specialty Hospital, Dubai, United Arab Emirates; ^3^ Division of Pediatric Cardiology, Department of Pediatrics and Adolescent Medicine, Children’s Heart Center, AUBMC, Beirut, Lebanon

**Keywords:** hypoplastic left heart syndrome, congenital heart disease, hybrid procedure, hybrid stage I, developing country

## Abstract

**Background:**

Hypoplastic left heart syndrome (HLHS) accounts for 2.6% of congenital heart disease and is an invariably fatal cardiac anomaly if left untreated. Approximately 33,750 babies are born annually with HLHS in developing countries. Unfortunately, the majority will not survive due to the scarcity of resources and the limited availability of surgical management.

**Aim:**

To describe and analyze our experience with the hybrid approach in the management of HLHS in a developing country.

**Methods:**

We performed a retrospective single-center study involving all neonates born with HLHS over five years at the Children's Heart Center at the American University of Beirut. The medical records of patients who underwent the hybrid stage 1 palliation were reviewed, and data related to baseline characteristics, procedure details and outcomes were collected to describe the experience at a tertiary care center in a developing country.

**Results:**

A total of 18 patients were diagnosed with HLHS over a five-year period at our institution, with male to female ratio of 1:1. Of those, eight patients underwent the hybrid stage I procedure. The mean weight at the time of the procedure was 3.3 ± 0.3 kg with an average age of 6.4 ± 4 days. The mean hospital length of stay was 27.25 days, with an interquartile range of 33 days. The cohort's follow-up duration averaged 5.9 ± 3.5 years. The surgical mortality was zero. Only one mortality was recorded during the interstage period between stage I and II and was attributed to sepsis. Notably, all surviving patients maintained preserved and satisfactory cardiac function with good clinical status.

**Conclusion:**

Our limited experience underscores the potential of developing countries with proper foundations to adopt the hybrid procedure for HLHS, yielding outcomes on par with those observed in developed countries. This demonstrates the viability of establishing a more balanced global landscape for children with congenital heart disease.

## 1 Introduction

Globally, approximately 1.5 million babies are born with congenital heart defects (CHD) annually (1, 2). Unfortunately, their survival prospects are closely linked to their geographical location and available medical resources. Tragically, 90% of CHD cases in low- and middle-income countries (LMICs) lack access to vital cardiac care (2–4), resulting in significantly elevated mortality and disability rates compared to their counterparts in high-income countries. The burden of CHD is particularly pronounced in regions with higher fertility rates and lower per capita income, representing 90% of births in the world's most economically disadvantaged areas (5).

Hypoplastic left heart syndrome (HLHS) constitutes 2.6% of all congenital heart diseases (6, 7), and thus, the estimated number of babies born with HLHS in LMICs each year is around 33,750. Sadly, most of these infants will not survive infancy due to the bleak prospects of receiving treatment.

HLHS is characterized by the underdevelopment of structures on the left side of the heart, including the mitral valve, left ventricle, aortic valve, and aortic arch. Before the 1980s, this complex malformation was linked to a staggering 95% mortality rate within the first month of life (8).

The introduction of the Norwood procedure in the early 1980s revolutionized the prognosis for these children. The majority now undergo a series of three surgical stages, enabling survival beyond the neonatal phase and into early adulthood. Neonatal treatment options for this condition include the Norwood operation or the hybrid procedure (9, 10). The hybrid procedure involves stenting the ductus arteriosus and banding the pulmonary branches to regulate pulmonary flow.

Managing infants diagnosed with HLHS in LMICs continues to face challenges due to the condition's complexity, poor surgical outcomes, and the necessity for specialized medical care. The management of HLHS raises multifaceted ethical, social, and economic concerns. Striking a balance between local health priorities, weighing the potential benefits of intervention against the risks, and allocating resources for one condition over others become intricate.

This study aims to elucidate the experience of employing the hybrid procedure for HLHS at a tertiary center in a developing nation. It encompasses the procedure's outcomes, patient follow-up, and associated mortality and morbidity rates. It also compares these findings to those in developed countries.

## 2 Materials and methods

This is a retrospective study that includes all neonates diagnosed with HLHS at the Children's Heart Center (CHC) at the American University of Beirut-Medical Center (AUBMC) in Lebanon between October 2013, and January 2018, inclusive. The CHC is a tertiary center in a developing country, and a major referral center in the Middle East and North Africa. The center treats annually around 2,000 patients, performs around 150–200 congenital heart surgeries and about 120–150 cardiac catheterizations. After institutional review board (IRB) approval, patients' data were collected from the AUBMC medical records. Collected data included demographics, surgical procedures, progress notes, imaging, cause of mortality, morbidity, and follow-up information.

**Figure 1:**
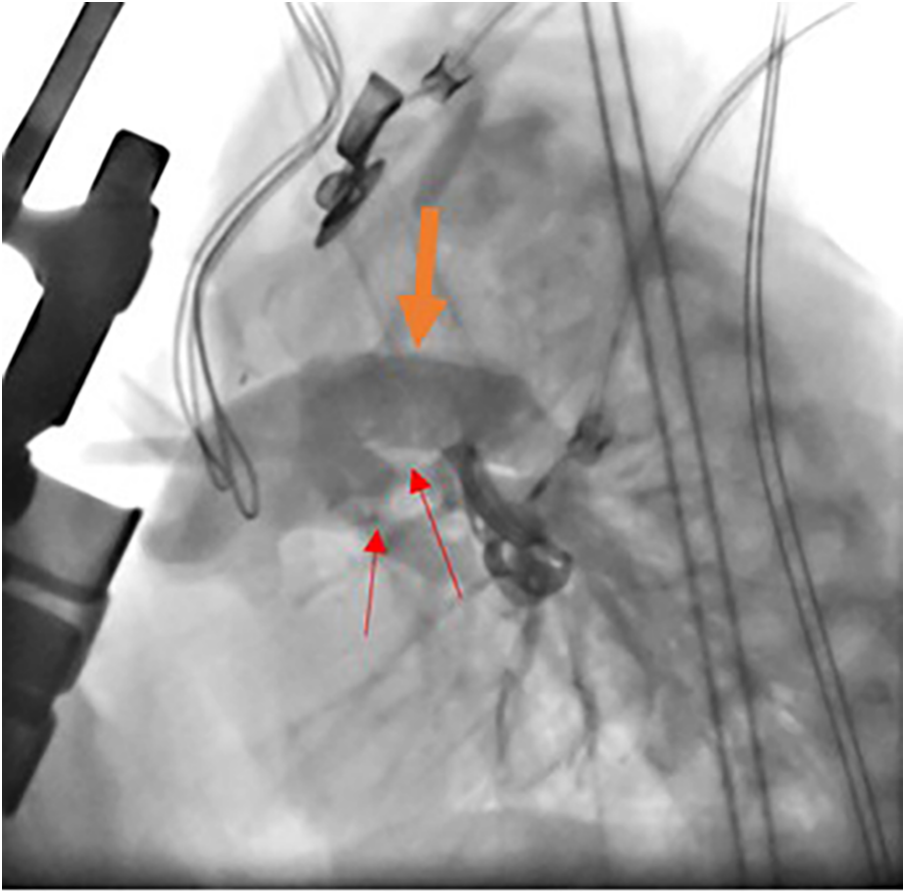
Catheterization during hybrid procedure. Angiographic study during stent deployment in hybrid stage I palliation. The modified lateral view shows the stent into the arterial duct (thick organ arrow) and bilateral banding of the PAs (thin red arrow).

**Figure 2:**
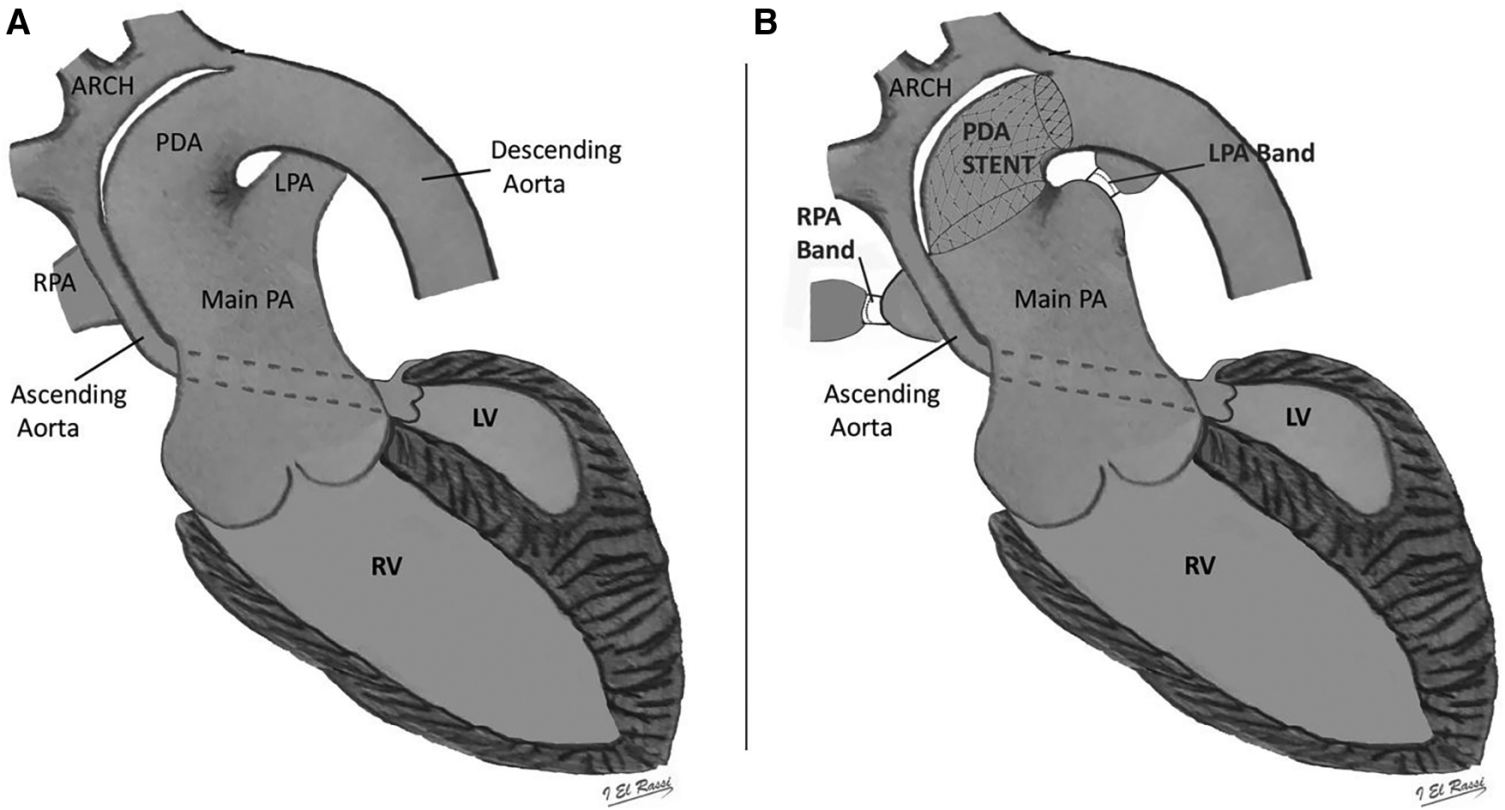
Schematic representation of the hybrid procedure. Panel **A** depicts hypoplastic left heart syndrome; Panel **B** is a schematic image of Hybrid stage 1 that shows stent into the arterial duct and bilateral banding of the LPA and RPA. PDA, patent ductus arteriosus; RPA, right pulmonary artery; LPA, left pulmonary artery; LV, left ventricle; RV, right ventricle.

The hybrid procedure is performed in the catheterization laboratory and includes the collaboration of a pediatric cardiac surgeon and a pediatric interventional cardiologist. Under general anesthesia, a median sternotomy is made, and the thymus is resected. Bilateral pulmonary artery (PA) banding is achieved using a 3.5 mm polytetrafluoroethylene graft, divided longitudinally, and wrapped around the branch pulmonary arteries for a width of approximately 3 mm. The bands are then secured to the adventitia of the main PA with 5-0 or 6-0 non-absorbable polypropylene sutures to avoid distal dislodging. The patient is usually partially heparinized, a purse-string suture is placed on the proximal main pulmonary artery, and a 6-Fr sheath is inserted. The ductal stent is deployed through the sheath under fluoroscopic guidance, achieving stage I palliation (Figure 1). We use standard-size balloon-expandable stents, 18 mm long and 8–9 mm in diameter, depending on availability. The used stents are either the Scuba™ Stent System or the Visio-Pro Stent System. The stent is placed in an appropriate position, confirmed by angiography. The saturation usually decreases by 8%–10% following the procedure to achieve a final saturation of 80%–85%, and the systolic BP increases by about 10 mm Hg. Balloon atrial septostomy is performed under echo guidance in a different setting. Figure 2 depicts a schematic representation of the hybrid procedure.

Patients underwent the second stage at an average age of 8.6 months (range 5–11 months), following clinical, echocardiographic and catheterization assessment. A repeat median sternotomy is performed under cardiopulmonary bypass and hypothermia. This stage includes the removal of the branch pulmonary arteries bands, resection of the PDA stent and dissection of the ductus arteriosus, creation of neoaorta through anastomosis of the ascending aorta and the main pulmonary artery, plasty of the pulmonary artery branches when needed, transection of the superior vena cava from the right atrium, and creation of the cavo-pulmonary connection.

Stage 3, completed at 3–5 years of age, is performed with an extracardiac fenestrated conduit connecting the inferior vena cava to the pulmonary arteries via a Gore-Tex conduit to form total cavo-pulmonary circulation.

## 3 Results

A total of 18 patients with the diagnosis of HLHS in the CHC at the AUBMC between October 2013 and January 2018 were identified and included in this retrospective cohort study.

**Figure 3:**
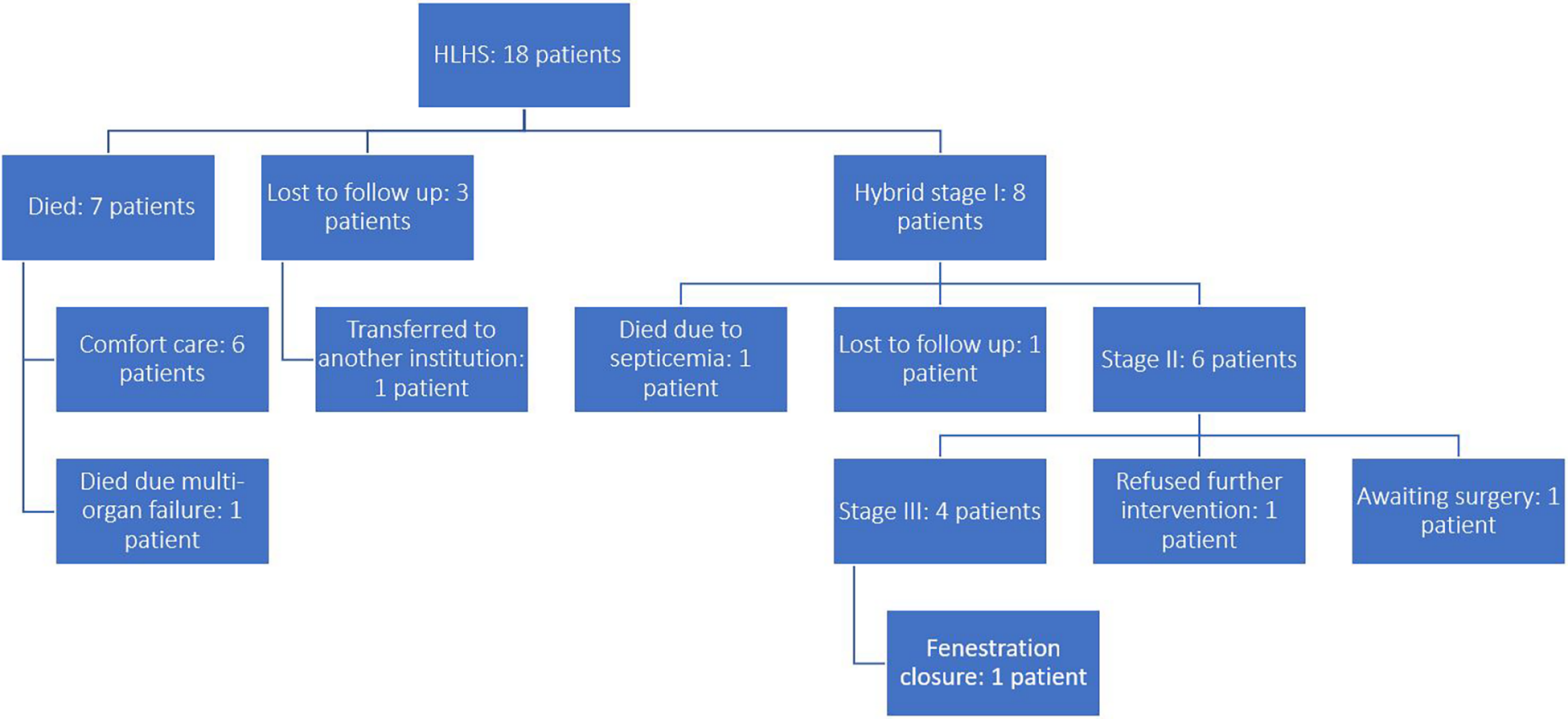
Flow chart of the patients in the study.

Females constituted 50% of the population. Only eight (44%) patients were operated on. Six (33%) patients were placed on comfort care after discussion with the parents regarding their medical condition, treatment options, risks and long-term prognosis; They survived on average until day of life eight. One patient (5.5%) underwent urgent bedside atrial septostomy, however later developed acute kidney injury, disseminated intravascular coagulation and shock, and eventually died at day of life 7. Another patient (5.5%) was transferred to another institution after being placed in comfort care and was lost to follow up thereafter. Two (11%) more patients presented to the outpatient clinic at day of life 10 and 38 respectively, however parents refused hospitalization and were lost to follow up. Figure 3 depicts the flow of patients in this study.

Among patients who underwent the hybrid stage I procedure, females constituted 62.5% of the cohort. The patients’ mean weight was 3.3 ± 0.3 kg, and the mean age was 6.4 ± 4 days at the time of the procedure. The mean length of stay at the hospital during the hybrid procedure was 27.25 days (IQR = 33 days). The average follow-up period for this cohort was 5.9 ± 3.5 years. Table 1 represents the demographic characteristics of these patients. Among the eight patients undergoing the hybrid procedure, two had mitral atresia along with hypoplastic aortic valve and ascending aorta, one patient had hypoplasia of mitral valve, aortic valve and ascending aorta with good left ventricular size, five patients had severe hypoplasia of the mitral valve, aortic valve, left ventricle and ascending aorta, and the last patient had hypoplastic aortic valve and ascending aorta.

**Table 1:** The demographic characteristics of patients who undergone the hybrid stage I procedure.

Variables	Mean ± SD
Female	5 (62.5%)
Age at the time of the procedure	6.4 ± 4 days
Weight	3.3 ± 0.3 kg
LOS Mean (IQR)	27.25 days (33 days)
Follow-up period	5.9 ± 3.5 years

LOS, length of stay; IQR, interquartile range.

Of the eight patients who completed the hybrid procedure, four underwent atrial septostomy, while two had atrial septectomy. Half of the patients (*n* = 4) of the patients had insertion of a 9 × 18 mm stent, while the other half had an 8 × 18 mm stent.

Three patients (37.5%) developed complications, which might be secondary to ductal steal. One patient developed bowel perforation of undetermined etiology and was managed surgically. Another patient developed transient renal failure with no other issues. The third patient developed unspecified nephrotic syndrome and did not require any intervention. These three patients had a mean length of stay of 44.3 ± 22.1 days, more than the mean of the collective cohort. The three patients left the hospital in good condition. None of the three patients died later.

Overall, the mortality rate for the hybrid procedure was zero. All patients (100%) were followed up with a preserved and good cardiac function. Only one death (12.5%) was recorded due to non-cardiac complications. This patient developed septicemia in the setting of an underlying metabolic disorder two months following his discharge from the hospital. Overall, the total mortality of the group at one month was 0%, at two months was 12.5%, and remained unchanged after following up for an average of 5 years. The survival rate after each operation is presented in Table 2.

**Table 2:** Hospital survival for operative surgeries.

Operation	*N* (%)	Survival (%)
Stage 1	8 (100%)	100%
BCPA	6 (75%)	100%
Fontan procedure	4 (50%)	100%
Additional surgeries: TV repair	2 (25%)	100%

BCPA, bidirectional cavopulmonary anastomosis.

Six of the eight patients had completed the Glenn procedure (75%) with no associated mortality. 25% (*n* = 2) of the patients underwent valvular repair of the tricuspid because of regurgitation, and none died. 50% of the patients (*n* = 4) underwent the Fontan procedure with 18 mm Gor-Tex^R^ and fenestration. One patient underwent successful device closure of a fenestrated Fontan. The rate of intervention involving the pulmonary arteries was high. Six patients required pulmonary artery plasty for single or bilateral pulmonary artery branches. Four patients had the plasty performed during stage 2. No stents were used.

Four patients did not undergo the Fontan procedure. One patient died two months after the hybrid procedure. Another patient did not undergo surgery since the parents were reluctant to do so after the benefits and risks were explained; the third patient is awaiting the Fontan surgery. The last patient was lost for follow-up after four months from the hybrid procedure and probably left for a neighboring country.

The surgical mortality rate for stage 2 and stage 3 palliation was 0%. Since the onset of the hybrid procedure, all patients were seen periodically. On their latest follow-up, all the patients demonstrated good ventricular systolic function and tricuspid valve competence as assessed by echocardiography.

## 4 Discussion

In this study we share our experience with the hybrid approach in palliating patients with HLHS. It is important to note that before 2013, no interventions were provided for patients with HLHS in our country. Therefore, our findings demonstrate the potential of the hybrid approach to be utilized in the management of neonates with HLHS, particularly in developing countries with restricted resources and limited surgical and peri-operative expertise.

Of the 18 patients with HLHS, eight patients successfully completed hybrid stage I. The long-term outcomes of our relatively small cohort, tracked over several years, unveiled a 5-year survival rate of 87.5% after completing the Norwood 2 comprehensive operation and the Fontan procedure. Remarkably, all surviving patients maintained a good functional cardiac status. Our eldest patient is a 10-year-old male, with echocardiographic findings revealing normal right ventricular function, alongside mild tricuspid regurgitation, during the latest follow-up. Five other patients are still being followed up at the pediatric cardiology outpatient clinic. Three patients, who completed stage 3, have good right ventricular systolic function, mild tricuspid regurgitation, and patent connections. One of them had device closure of the fenestrations, and another patient has mild to moderate aortic regurgitation. The patient, whose parents opted not to proceed to the third stage, exhibits mildly diminished right ventricular function, moderate tricuspid regurgitation, and mild neoaortic regurgitation. The last patient, who is currently awaiting the third stage, has good right ventricular function with mild to moderate tricuspid regurgitation.

Our current experience shows a 0% mortality rate of stage 2 and 3, surpassing the survival rates described in the literature. However, these findings may be influenced by the limited size of the study population. Nevertheless, the mortality rates of comprehensive stage 2 reported by large studies in the literature are still reassuring and promising, estimated at 4%–9% (11). Additionally, other variables, including the low birth weight, the presence of associated congenital anomalies, and concomitant non-cardiac morbidities, which might contribute to procedural failure and increased complications during stage 2, are absent in our population. Most importantly, our high success rate is attributed to the effective communication and coordination between the interventional and surgical teams. Furthermore, it is crucial to highlight that our center performs around 200 congenital heart surgeries annually, necessitating an effective multidisciplinary approach involving surgical, pediatric, cardiology, and intensive care teams and requiring solid peri-operative care. Thus, our robust and impactful experience in post-operative and pediatric intensive care management significantly contributed to improved peri-operative and overall outcomes.

Therefore, despite operating with a modest volume, our surgical mortality and length of stay align with those reported in developed countries. Our experience underscores that successful HLHS surgical programs can be established in LMICs if a suitable foundation is in place, yielding favorable outcomes. Our findings offer valuable insights into the applicability and viability of these hybrid procedures in developing nations.

Therefore, despite operating with a modest volume, our surgical mortality and length of stay align with those reported in developed countries. Our experience underscores that successful HLHS surgical programs can be established in LMICs if a suitable foundation is in place, yielding favorable outcomes. Our findings offer valuable insights into the applicability and viability of these hybrid procedures in developing nations.

HLHS presents as a uniformly lethal cardiac anomaly if left untreated. The introduction of the Norwood procedure in the early 1980s marked a pivotal moment in HLHS treatment. The hybrid procedure later emerged as a groundbreaking strategy to manage neonates with high-risk HLHS. The concept was first introduced in 1993 by Gibbs et al., when bilateral pulmonary artery bands were placed surgically, followed by the placement of a stent in the ductus arteriosus (12). This has opened the door for additional innovative interventions to address neonates with HLHS who are not eligible for the Norwood procedure. Subsequently, Akintuerk et al. modified the procedure and highlighted its cardinal role in providing a stepwise approach to palliate HLHS (13). However, the technique was still performed in two different settings: the operating room and the catheterization lab. This hybrid strategy continued to evolve until it was first introduced as a single procedure consisting of the placement of PA bands and PDA stent simultaneously, introducing the first true hybrid procedure (14). Following these milestones, the experience with hybrid procedure has expanded and improved significantly (15–17). However, despite all the technical development and the fact that outcomes for Hybrid I and Norwood procedures have demonstrated comparability, the Norwood procedure remains more prevalent in developing countries.

In our context, adopting the hybrid approach stemmed from local expertise, involving collaboration between the local pediatric cardiac surgeon and the pediatric interventional cardiology team. Moreover, the hybrid procedure was favored as it circumvents open-heart surgery and cardiopulmonary bypass and is feasible within a cardiac catheterization suite. By avoiding cardioplegia and cardiopulmonary bypass, the hybrid approach reduces procedural duration and hospital stay, and holds significantly lower financial burden (2). Notably, when evaluating risk-adjusted outcomes, there was no significant disparity between the primary Norwood and bilateral pulmonary artery banding groups in higher-volume institutions (4).

Besides, surgical outcomes have shown notable improvement over subsequent decades. The mortality rate for stage 1 palliation decreased from 40% to 30% within a decade. A study by Mascio et al. elucidated that a total of 1,663 Norwood procedures had a survival rate demonstrating a positive trajectory, with mortality rates dropping from 40.4% in the mid-80s to a plateau of 15.7% in the mid-2010s (18). According to the 2020 Harvest STS CHSD feedback report, a mortality rate of 12.4% with an interquartile range of 6.5%–16.2%, accompanied by average postoperative hospital stay of 58.7 days and an interquartile range of 43.7–66.9 days was reported in patients following the Norwood procedure (19).

Surgical treatment for HLHS in limited-resource countries has remained uncommon, with only a handful of centers in LMICs undertaking HLHS surgery. These efforts have primarily focused on Norwood stage 1 surgery, demonstrating a 41% survival rate after the initial stage. A few instances of utilizing the hybrid stage 1 procedure are documented in the Philippines (20) and India (21).

The hybrid stage I palliative procedure is a logical alternative to traditional Norwood or Sano operations for neonates with HLHS in LMICs. Challenges such as limited experience with cardiopulmonary bypass, circulatory arrest in neonates, and elevated costs contribute additional risks to surgeries in resource-constrained settings. Therefore, hybrid approaches yield good outcomes when resources permit, irrespective of geographical location. HLHS imposes a substantial financial burden, entailing a series of approximately three procedures: Norwood, Glenn, and Fontan. Dean et al. revealed mean operation costs of $214,680, $82,174, and $79,549, respectively (22). Adopting the hybrid 1 procedure could alleviate the expense of the initial operation. Nevertheless, challenging stages and interstage periods following the hybrid procedure should be expected. Indeed, the risks associated with palliation are not eradicated but are rather moved from stage 1 to later stages. Choosing the hybrid approach holds high rates of reintervention along with their associated risk of complications and mortality (16, 17). This is particularly important during comprehensive stage 2 in the setting of elevated pulmonary pressure or the presence of poorly confluent pulmonary vessels. Besides, heart transplant might be unavoidable in several patients. Close monitoring during the interstage period is also essential, however, might not always be feasible. Several patients were reported to experience sudden death during the interstage period while at home (16). Other significant concerns include retrograde aortic arch obstruction and the fate of the branch pulmonary arteries which might face significant distortion. The reconstruction of pulmonary arteries is challenging, as achieving reasonable confluence is crucial for subsequent successful Fontan. The need for interventions in the branch pulmonary artery is variable and can reach high rates, reported at 18%–86%, which exceeds the rate of intervention required after Norwood (11, 16, 23, 24).

Our experience at a developing country's tertiary center is encouraging, showing outcome measures comparable to those in developed countries. Specifically, our in-house surgical mortality for stage I hybrid procedures was zero, and our survival rate aligned with that of developed nations. Although limited by the humble population size, this experience with the hybrid approach in managing HLHS can pave the way for further utilization of hybrid interventions in developing countries. As discussed by our colleagues, the hybrid approach can provide the best out of transcatheter and surgical techniques in treating several congenital heart lesion, ranging from defect closure to managing valvulopathy (25). Therefore, the hybrid management of HLHS can be used as an example to manage other conditions in developing countries that suffer from the lack of expertise and resources. This partnership can shape and influence the future of pediatric and congenital cardiology.

It's important to acknowledge the limitations of our cohort study, including its small sample size and limited patient pool. Another limitation is attributed to the inherent retrospective nature of the study. Besides, one of the patients was lost to follow-up after undergoing the hybrid procedure, leaving 12.5% of the study population with an unknown fate. In addition, only 50% of the study population have completed the Fontan, placing restrictions on the feasibility of addressing stage 3 outcomes and long-term follow-up data. However, one patient is expected to complete stage 3 in the coming few months.

While the aspiration to provide optimal care for HLHS patients in developing or resource-limited countries is admirable, challenges tied to medical expertise, facilities, postoperative care, and long-term follow-up must be considered. The feasibility, safety, and ethical considerations of treating intricate conditions like HLHS in resource-constrained settings warrant careful assessment.

Parents and physicians confront arduous decisions regarding appropriate therapeutic interventions for infants with HLHS (8). To foster a more equitable landscape for children with congenital heart disease worldwide and address global disparities in pediatric cardiac care, we share our experience treating HLHS infants at a tertiary center in a developing country.

This limited experience showcases that adequately staffed developing countries can opt for the modified hybrid procedure for HLHS, yielding outcomes on par with those in developed countries. It underscores the feasibility of establishing a more equitable landscape for children with congenital heart disease worldwide.

## 5 Conclusion

The hybrid palliation stage 1 Norwood is a promising alternative for open heart surgery in the setting of a tertiary center in a developing country with no in-hospital mortality. Its implementation heavily depends on the developing country and its institutions, whether it has solid infrastructure alongside a collaborative group of medical professionals to deliver the appropriate care. To our knowledge, very few studies explore the matter of HLHS in developing countries. Additional studies are needed to understand better the status of the management of HLHS in developing countries.

## Data Availability

The original contributions presented in the study are included in the article/Supplementary Material, further inquiries can be directed to the corresponding authors.
